# Temporal profile of body temperature in acute ischemic stroke: relation to infarct size and outcome

**DOI:** 10.1186/s12883-016-0760-7

**Published:** 2016-11-21

**Authors:** Marjolein Geurts, Féline E. V. Scheijmans, Tom van Seeters, Geert J. Biessels, L. Jaap Kappelle, Birgitta K. Velthuis, H. Bart van der Worp, on behalf of the DUST investigators

**Affiliations:** 1 Department of Neurology and Neurosurgery, Brain Center Rudolf Magnus, University Medical Center Utrecht, Utrecht, The Netherlands; 2 Department of Radiology, University Medical Center Utrecht, Utrecht, The Netherlands

**Keywords:** Hypothermia, Cerebral infarction, Body temperature

## Abstract

**Background:**

High body temperatures after ischemic stroke have been associated with larger infarct size, but the temporal profile of this relation is unknown. We assess the relation between temporal profile of body temperature and infarct size and functional outcome in patients with acute ischemic stroke.

**Methods:**

In 419 patients with acute ischemic stroke we assessed the relation between body temperature on admission and during the first 3 days with both infarct size and functional outcome. Infarct size was measured in milliliters on CT or MRI after 3 days. Poor functional outcome was defined as a modified Rankin Scale score ≥3 at 3 months.

**Results:**

Body temperature on admission was not associated with infarct size or poor outcome in adjusted analyses. By contrast, each additional 1.0 °C in body temperature on day 1 was associated with 0.31 ml larger infarct size (95% confidence interval (CI) 0.04–0.59), on day 2 with 1.13 ml larger infarct size(95% CI, 0.83–1.43), and on day 3 with 0.80 ml larger infarct size (95% CI, 0.48–1.12), in adjusted linear regression analyses. Higher peak body temperatures on days two and three were also associated with poor outcome (adjusted relative risks per additional 1.0 °C in body temperature, 1.52 (95% CI, 1.17–1.99) and 1.47 (95% CI, 1.22–1.77), respectively).

**Conclusions:**

Higher peak body temperatures during the first days after ischemic stroke, rather than on admission, are associated with larger infarct size and poor functional outcome. This suggests that prevention of high temperatures may improve outcome if continued for at least 3 days.

## Background

Acute ischemic stroke is a devastating disease, leaving more than half of patients with a poor functional outcome [1]. High body temperatures in the early stage after ischemic stroke have consistently been associated with poor functional outcome [2–13]. Preclinical studies suggest that hyperthermia increases metabolic demands, release of neurotransmitters, free-radical production and breakdown of the blood–brain barrier after cerebral ischemia, hereby increasing cell death and infarct volume [14].

The association between body temperature and infarct size in patients with ischemic stroke is however still controversial, mainly when it comes to the temporal profile of this association. Two studies did not find a relation with body temperatures on admission [4] or after 6–12 h [15]. One study did show an association between infarct size and body temperature on admission, [11] and two between infarct size and body temperature at 24 h [3, 6]. Temperature assessment in all studies was limited to the first 24 h after stroke onset.

The temporal profile of the association between body temperature and functional outcome or death also show inconsistent results. Several studies have suggested that this is limited to body temperatures on admission or during the first day, [3, 5, 6, 10, 11, 13] whereas others have found that this relation persists for up to one week [2, 7–9, 12]. These inconsistencies may be attributed to differences in study designs and populations, [8] for example related to the time of admission, [13] the definition of a poor outcome outcome, [11] and selection of patient populations [3, 10].

In this study, we assessed the temporal profile of the relation between body temperatures during the first three days after ischemic stroke and infarct size and functional outcome.

## Methods

This is a substudy of the Dutch acute STroke study (DUST). Patients older than 18 years were included between May 2009 and August 2013 if they had symptoms suspected to be caused by ischemic stroke. Inclusion criteria were symptom duration <9 h, and National Institutes of Health Stroke Scale (NIHSS) ≥2, or ≥1 if intravenous thrombolysis with recombinant tissue type plasminogen activator (IV-rtPA) was indicated. Patients were not eligible if another diagnosis on non contrast CT (NCCT) such as intracranial hemorrhage explained the symptoms. Patients with an unknown onset time were included if the elapsed time between the time they were last seen without symptoms and imaging was <9 h [16].

We selected patients enrolled at the five of 14 DUST study centers that had included over 100 patients. Tympanic or rectal temperatures over the first 72 h after stroke onset were retrospectively collected from patients’ charts by one single investigator (FEVS), who was blinded for outcome measures. For each patient, we recorded the mean body temperature and the peak body temperature (highest body temperature) on days one to three after admission. Body temperature on admission was defined as the first recorded body temperature within six hours after admission; day one as the first 24 h after stoke onset, day two as 24 to 48 h, and day three as 48 to 72 h after stroke onset. Patients were included if at least one body temperature was recorded.

Infarct size was measured three (± two) days after symptom onset. The default follow-up imaging modality was non contrast CT (NCCT) after 3 days or at the time of clinical deterioration or earlier discharge. Follow-up MRI was used if this had been performed for clinical reasons instead of NCCT. Infarct volume was obtained by manually delineating the hypodense infarcted area(s) on axial NCCT slices and hyper-intense area(s) on axial DWI slices on MRI. The surface of these area(s) was subsequently multiplied by the slice thickness to obtain the infarct volume [16]. Patients with no visible infarct on follow-up scan were included in the analyses with an infarct volume of 0 ml. Functional outcome was measured with the modified Rankin Scale (mRS) at 90 days by a trained research nurse or neurologist. Poor outcome was defined as mRS ≥3.

The primary outcome measure was infarct volume (ml) at 3 days. The relation between each additional 1.0 °C in body temperature and infarct size was calculated by means of linear regression, and the relation between body temperatures and functional outcome with Poisson regression analysis with a robust error. The relation was expressed as regression coefficient (B) or relative risk (RR) with corresponding 95% confidence interval (CI), respectively. We adjusted for age, sex, previous stroke, hypertension, diabetes mellitus, current smoking, treatment with intravenous alteplase, intra-arterial treatment, and National Institutes of Health Stroke Scale (NIHSS) score on admission, with backward stepwise regression with 0.10 alpha levels of removal. Potential confounders were selected on basis of known associations with the outcome. We considered a *p*-value ≤0.05 significant.

## Results

**Fig. 1:**
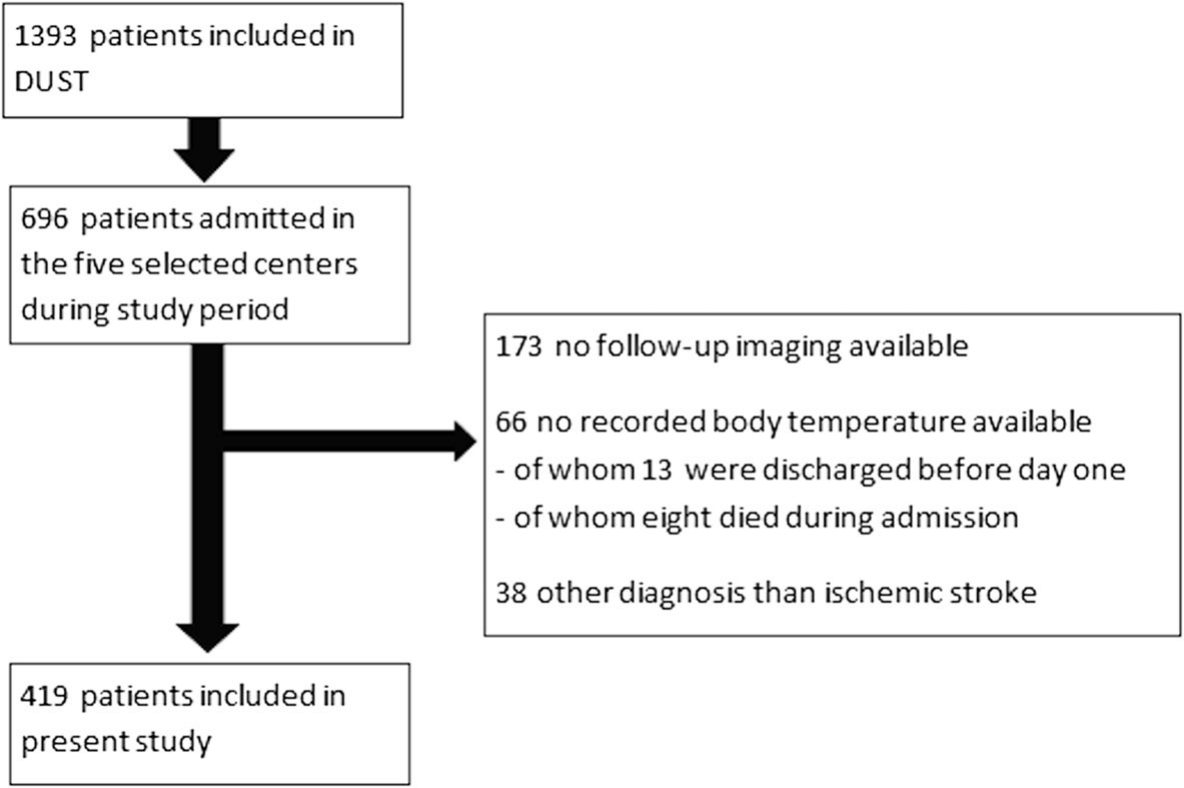
Flow of patients through this study

Of 1393 patients included in DUST, 696 were included in the five selected centers. We included 419 of these patients for the present study, after excluding 173 patients without follow-up imaging, 66 without a recorded body temperature available and 38 with an other diagnosis than ischemic stroke (Fig. 1).

The mean age of the patients was 66 years (SD 13); 256 (61%) were male. Additional patient characteristics are presented in Table 1. Follow-up imaging was performed with CT (95%) or MRI (5%). Patients without follow-up imaging were older (70 vs 66 years, *p* = 0.001), were more often men, and had a higher median NIHSS score on admission (7 vs 6, *p* = 0.02; Table 1).

**Table 1:** Patient characteristics

	Included patients (*n* = 419)	Patients without follow-up imaging (*n* = 173)	*p*-value
Age (years)	66 (13)	70 (15)	0.001
Men	256 (61)	84 (49)	0.01
Body temperature on admission (°C)	36.7 (0.6)	36.7 (0.8)	0.94
NIHSS on admission	6 (10)	7 (10)	0.02
Hypertension	206 (49)	102 (59)	0.25
Diabetes mellitus	65 (16)	23 (13)	0.49
Current smoking	122 (29)	38 (22)	0.39
Previous stroke	88 (21)	43 (25)	0.38
TOAST			0.66
Large-artery atherosclerosis	130 (31)	51 (30)	
Cardioembolism	79 (19)	33 (19)	
Small vessel disease	49 (12)	16 (9)	
Other	24 (6)	4 (9)	
Unknown	137 (32)	58 (33)	
Posterior circulation stroke	14 (3)	2 (3)	0.67
Treatment with intravenous alteplase	249 (59)	112 (65)	0.29
Intra-arterial treatment	26 (6)	11 (6)	0.94
Poor outcome (mRS ≥3)	144 (34)	71 (41)	0.11

Data are n (%), median (range), median (interquartile range (IQR)) or mean (standard deviation (SD)) where appropriate. NIHSS, National Institutes of Health Stroke Scale; TOAST, Trial of Org 10172 in Acute Stroke Treatment classification; mRS, modified Rankin Scale

**Fig. 2:**
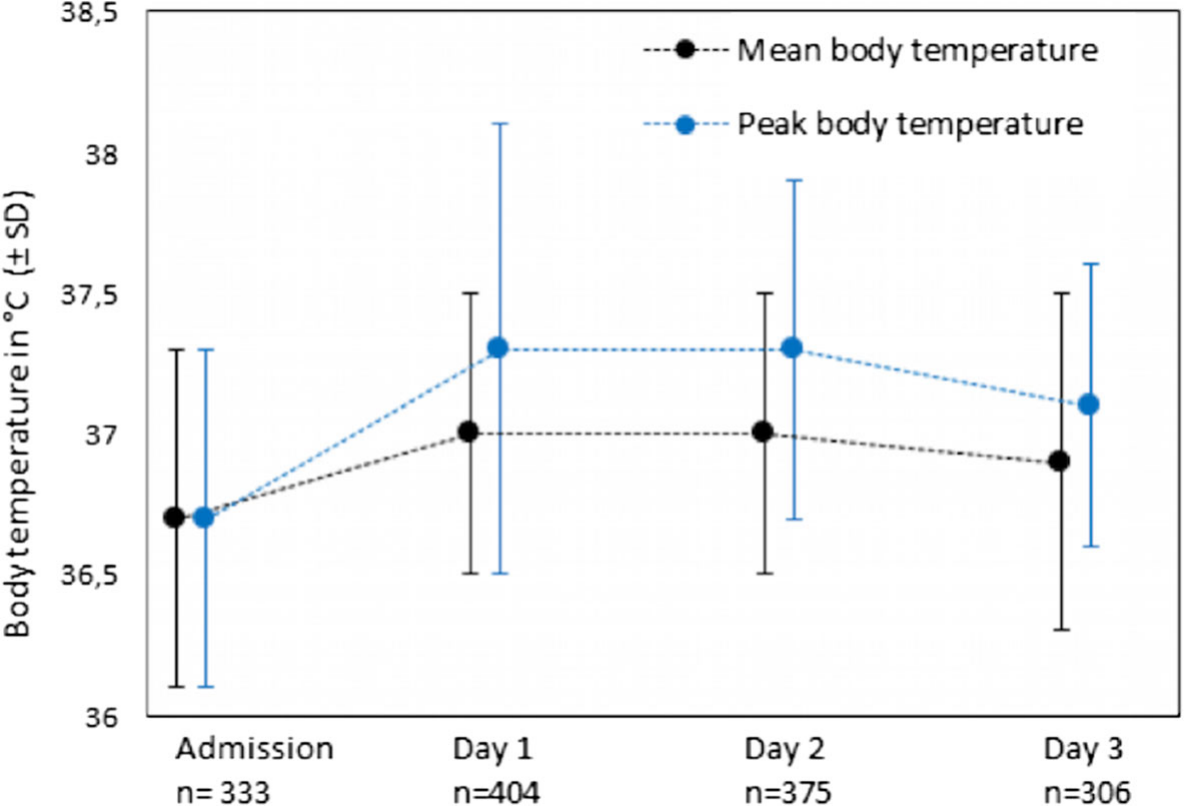
Course of body temperatures in the first 3 days after stroke onset

At follow-up, median infarct volume was 1.5 ml (range, 0–500 ml) in the total study population of 419 patients. There were 131 (31%) patients without a visible infarct on follow up imaging, i.e. an infarct volume of 0 ml. The mean body temperatures during the first 3 days are presented in Fig. 2.

A total number of 406 patients had at least one body temperature recorded at day one, 376 patients at day two, and 308 patients at day three. The mean peak body temperature on day one was 37.3 °C (SD 0.8), on day two 37.3 °C (SD 0.6), and on day three 37.1 °C (SD 0.5; Fig. 1). Mean and peak body temperatures were higher at days one, two and three than on admission (*p* < 0.001 for all days).

**Fig. 3:**
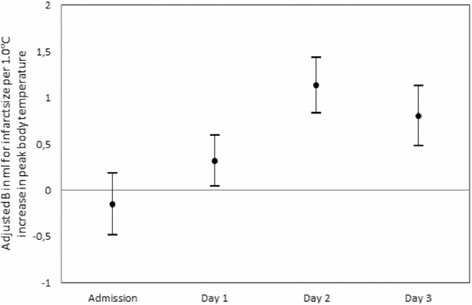
Relation between infarct size and peak body temperature

**Fig. 4:**
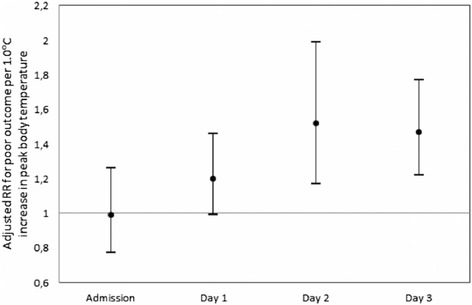
Relation between poor functional outcome and peak body temperature

Higher peak body temperatures on days one, two and three after stroke onset were associated with larger infarct size. In adjusted linear regression analyses, each additional 1.0 °C in body temperature on day 1 was associated with 0.31 ml larger infarct size (95% CI, 0.04–0.59), on day 2 with 1.13 ml larger infarct size (95% CI, 0.83–1.43), and on day 3 with 0.80 ml larger infarct size (95% CI, 0.48–1.12) (Fig. 3, Table 2). Peak body temperatures on days two and three were also associated with a poor outcome (Fig. 4, Table 2). With every additional 1.0 °C in peak body temperature on days two and three, the risk of a poor outcome was 52% (95% CI, 17–99%) and 47% (95% CI, 22–77%) larger, respectively. Body temperature on admission was neither related to infarct size nor to functional outcome. Mean body temperatures at days one, two and three were, after adjustment, neither associated with infarct size nor with poor functional outcome.

**Table 2:** The relation between body temperature and infarct size

	Infarct size at day 3 (±2)			Functional outcome at 90 days		
	After adjustment^a^			After adjustment^a^		
	B	95%CI	*P*	RR	95%CI	*P*
Body temperature on admission *n = 333*	−0.15	−0.49–0.18	0.38	0.99	0.77–1.26	0.90
Peak body temperature on day 1 *n = 404*	0.31	0.04–0.59	0.02	1.20	0.99–1.46	0.06
Mean body temperature on day 1 *n = 404*	0.09	−0.41–0.60	0.71	1.38	0.84–2.28	0.21
Peak body temperature on day 2 *n = 375*	1.13	0.83–1.43	<0.001	1.52	1.17–1.99	0.002
Mean body temperature on day 2 *n = 375*	−0.27	−0.78–0.26	0.33	0.74	0.33–1.64	0.46
Peak body temperature on day 3 *n = 306*	0.80	0.48–1.12	<0.001	1.47	1.22–1.77	<0.001
Mean body temperature on day 3 *n = 306*	0.40	−0.18–0.97	0.17	1.64	0.83–3.25	0.16

B, regression coefficient in ml per additional 1.0 °C in body temperature; CI, confidence interval

^a^Adjusted for age, sex, previous stroke, hypertension, diabetes mellitus, current smoking, treatment with intravenous alteplase, intra-arterial treatment and National Institutes of Health Stroke Scale score on admission

### Subgroup analyses

In a post-hoc subgroup analysis of 288 patients with a visible infarct on follow-up imaging, median infarct volume was 9.6 ml (range, 0.2–500). Results were essentially the same (Table 3). In a post-hoc subgroup analysis of 398 patients with CT as follow-up imaging modality, results were essentially the same (data not shown).

**Table 3:** Relation between body temperature and infarct size in patients with an infarct of >0 ml

	Infarct size at day 3 (±2)			Functional outcome at 90 days		
	After adjustment^a^			After adjustment^a^		
	B	95%CI	*P*	RR	95%CI	*P*
Body temperature on admission *n = 202*	0.04	−0.32–0.40	0.83	1.10	0.83–1.48	0.53
Peak body temperature on day 1 *n = 273*	0.26	−0.04–0.57	0.09	1.19	0.95–1.48	0.13
Mean body temperature on day 1 *n = 273*	0.28	−0.43–0.99	0.44	1.23	0.50–3.03	0.65
Peak body temperature on day 2 *n = 244*	0.87	0.53–1.22	<0.001	1.43	1.07–1.91	0.02
Mean body temperature on day 2 *n = 244*	0.06	−0.59–0.72	0.85	0.64	0.27–1.05	0.31
Peak body temperature on day 3 *n = 175*	0.62	0.27–0.97	0.001	1.45	1.14–1.84	0.002
Mean body temperature on day 3 *n = 175*	0.90	0.22–1.60	0.01	1.49	0.73–3.10	0.28

B, regression coefficient in ml per additional 1.0 °C in body temperature; CI, confidence interval

^a^Adjusted for age, sex, previous stroke, hypertension, diabetes mellitus, current smoking, treatment with intravenous alteplase, intra-arterial treatment and National Institutes of Health Stroke Scale score on admission

## Discussion

This study shows that in patients with acute ischemic stroke, higher peak body temperatures on days one, two and three after stroke onset are associated with larger infarct size. Peak body temperatures on days two and three were also associated with poor functional outcome after 3 months. Body temperature at admission was neither related to infarct size nor to functional outcome.

This is the first study that assesses the temporal profile of the association between body temperature and infarct size in the first days after stroke.

High body temperature after stroke may be the result of infections, which are frequent and have also been associated with poor functional outcome. However, no source of infection could be found in almost half of the hyperthermic patients in a previous study [8]. Fever could also be the result of an inflammatory response of the body to the infarcted tissue. Additionally, in the first days after stroke temperature-dependent processes which lead to increased extracellular edema, infarct swelling, and restricted capillary flow in the ischemic tissue, can increase ischemic damage [8]. Although increased body temperature is often thought to be a reflection of extensive cerebral damage, we found an association between increased body temperature and poor functional outcome that was independent of baseline stroke severity.

If the relation between higher body temperatures and larger infarct volumes and poor functional outcome is at least partially causal, our findings suggest that a reduction in body temperature up to 3 days after stroke may reduce infarct size and improve functional outcome. In a post-hoc subgroup analysis of the randomized Paracetamol (Acetaminophen) In Stroke (PAIS) trial, treatment with paracetamol for 3 days was associated with an improvement in functional outcome at 3 months in patients with a baseline body temperature of 37.0 °C or above, [17] supporting the causal relationship between body temperature and functional outcome.

Our study has limitations. First, we had to exclude 173 patients because of lack of follow-up scans, and of the remaining patients we excluded 10% without any recorded temperature. Up to 27% of included patients did not have temperature measurements on one of the 3 days. We used tympanic and rectal temperatures interchangeably, whereas values may differ between those methods. In addition, patients in our study may have been treated with antipyretics, which could have affected temperature measurements and would underestimate the number of patients with high body temperatures. However, by assessing the peak body temperatures rather than mean temperatures, we aimed to assess body temperatures before administration of antipyretics. This may also explain our finding that mean body temperatures were not related to infarct size or functional outcomes. Of the included patients, one third had no visible infarct on follow-up CT. One could argue that these patients did not suffer from cerebral ischemia. However, in subgroup analysis including patients with a visible infarct results were essentially the same. We included patients without a visible infarct on follow-up imaging in this substudy to ensure the association we assess applies to all patients with the clinical diagnosis of stroke, including the small strokes and patients that recover completely. Infarct size was measured on either CT or MRI. As the default follow-up modality was CT, it is possible that some smaller infarcts were not detected. However, in subgroup analysis including only patients with CT as follow-up modality, results were essentially the same.

With a median NIHSS of 6 on admission, included patients had relatively milder strokes than excluded patients (median NIHSS of 7). Our data may differ in a selection of patients with severe stroke. The time between stroke onset and first measurement of temperature was not predefined in the DUST study protocol. Therefore, the variation between time from stroke onset to first recorded body temperature might have affected our results. We did not have data on the occurrence of infections in our population and could therefore not analyze their relationship with hyperthermia. We present results per 1.0 °C, which results in wide confidence intervals.

As a result of small patient numbers in extreme body temperature categories, this study is insufficient to detect associations at body temperatures lower than 35.5 °C or higher than 38.5 °C.

## Conclusions

In conclusion, we found that higher body temperatures in the first days after ischemic stroke, rather than on admission, are associated with larger infarct size and poor functional outcome. Our findings suggest that prevention of high temperatures in clinical trials may improve outcome if continued for at least three days.

Guidelines recommend the use of antipyretics for febrile patients with stroke, but do not provide a time window [18]. In the randomised Paracetamol (Acetaminophen) In Stroke (PAIS) trial, treatment of patients with a baseline body temperature of 37 °C or above with high-dose paracetamol, started within 12 h of stroke onset and continued for 3 days, resulted in a temperature reduction of just 0.3 °C at 24 h, but also in an improved outcome at 3 months [17]. A large phase III trial on the effect of induced hypothermia after stroke is ongoing, cooling patients 12 to 24 h after their stroke [19]. Future clinical trials should further assess the effect of preventing fever or inducing hypothermia up to at least 3 days after stroke.

## Abbreviations

CI: Confidence interval
DUST: Dutch acute STroke study
mRS: modified Rankin Scale
NCCT: Non contrast CT
NIHSS: National Institutes of Health Stroke Scale
PAIS: Paracetamol (Acetaminophen) In Stroke
RR: Relative risk
